# Social effects on fruit fly courtship song

**DOI:** 10.1002/ece3.4759

**Published:** 2018-12-10

**Authors:** Lucas Marie‐Orleach, Nathan W. Bailey, Michael G. Ritchie

**Affiliations:** ^1^ School of Biology, Centre for Biological Diversity University of St Andrews St Andrews UK

**Keywords:** acoustic signals, behavioral plasticity, reproductive isolation, social learning, speciation

## Abstract

Courtship behavior in *Drosophila* has often been described as a classic innate behavioral repertoire, but more recently extensive plasticity has been described. In particular, prior exposure to acoustic signals of con‐ or heterspecific males can change courtship traits in both sexes that are liable to be important in reproductive isolation. However, it is unknown whether male courtship song itself is socially plastic. We examined courtship song plasticity of two species in the *Drosophila melanogaster *subgroup. Sexual isolation between the species is influenced by two male song traits, the interpulse interval (IPI) and sinesong frequency (SSF). Neither of these showed plasticity when males had prior experience of con‐ and heterospecific social partners. However, males of both species produced longer bursts of song during courtship when they were exposed to social partners (either con‐ or heterospecific) than when they were reared in isolation. *D. melanogaster* carrying mutations affecting short‐ or medium‐term memory showed a similar response to the social environment, not supporting a role for learning. Our results demonstrate that the amount of song a male produces during courtship is plastic depending on the social environment, which might reflect the advantage of being able to respond to variation in intrasexual competition, but that song structure itself is relatively inflexible, perhaps due to strong selection against hybridization.

## 1 INTRODUCTION

The role of behavioral flexibility in evolution is controversial (Bailey, Marie‐Orleach, & Moore, 2018; Price, Qvarnstrom, & Irwin, 2003). In the context of speciation, the evolution and maintenance of reproductive barriers between species can be strongly affected if mate preferences are learned or otherwise influenced through social experience (Dukas, 2013; Irwin & Price, 1999; Servedio & Dukas, 2013; Verzijden et al., 2012). For example, experiencing heterospecifics is often found to strengthen conspecific mating preferences (delBarco‐Trillo, McPhee, & Johnston, 2010; Dukas, 2008; Fincke, Fargevieille, & Schultz, 2007; Kozak & Boughman, 2009; Magurran & Ramnarine, 2004). It is also critical to understand how social experience affects sexual signals upon which mating decisions are based (Verzijden et al., 2012), but signal plasticity tends to receive less study. Of the studies that have tested this, some have found extensive learning effects in signaling, such as birds or sea mammals which learn song (Garland et al., 2011; Slabbekoorn & Smith, 2002), and this may accelerate speciation under some circumstances (Verzijden et al., 2012). In insects, song is more likely to be assumed to be innate, although social experience has been shown to influence signaling and reproductive tactics in several species (Bailey, Gray, & Zuk, 2010; Rebar, Barbosa, & Greenfield, 2016), and more broadly social learning can influence sexual isolation (Svensson, Eroukhmanoff, Karlsson, Runemark, & Brodin, 2010).

In species of the *Drosophila melanogaster* subgroup, several sexual traits show social plasticity (Schneider, Atallah, & Levine, 2017), including cuticular hydrocarbon profiles (Krupp et al., 2008), mate choice (Dukas, 2005; Mery et al., 2009), mating success (Billeter, Jagadeesh, Stepek, Azanchi, & Levine, 2012), and ejaculate characteristics (Garbaczewska, Billeter, & Levine, 2013; Sirot, Wolfner, & Wigby, 2011; Wigby et al., 2009). Recently, both male and female responses to song were shown to be influenced by social experience transmitted through species‐specific male courtship song itself (Li, Ishimoto, & Kamikouchi, 2018). Li et al. (2018) found that both female song discrimination and a measure of male sexual arousal, “chaining,” were influenced, or tuned, more extensively following exposure to song carrying conspecific‐like IPIs than hetrospecific‐like song, suggesting an important role for social experience in the auditory channel in causing plastic behavioral responses to auditory social information.

**Figure 1:**
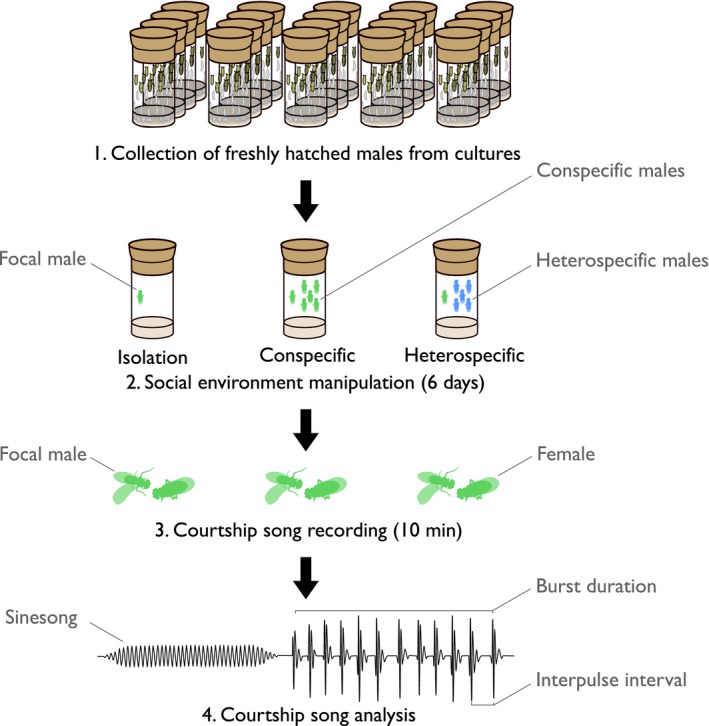
Experimental procedure to test the social plasticity of courtship song. We manipulated the social environment by raising focal males for 6 days either in isolation, with five conspecific males, or with five heterospecific males. We exposed focal males to virgin females for 10 min, and recorded the courtship song. We assessed the key parameters of courtship songs: interpulse interval (IPI), sinesong frequency (SSF), and pulse song burst duration

Here we focused on whether courtship song itself is socially plastic. Song is produced by wing vibrations, and two distinct parameters of this important mating signal, the interpulse interval (IPI) of pulse song and frequency of sinusoidal sinesong (SSF; Figure 1), are well‐characterized targets of female mate choice (Ewing & Bennet‐Clark, 1968; von Schilcher, 1976). Courtship song is species specific (Cowling & Burnet, 1981), evolves rapidly (Ritchie & Gleason, 1995), and females are more receptive when they are stimulated by conspecific song (Ritchie, Halsey, & Gleason, 1999). Genes with a large influence on song characteristics have been identified and manipulated (Ding, Berrocal, Morita, Longden, & Stern, 2016; Neville et al., 2014; Wheeler et al., 1991), and fly song is in many ways a classic study system for behavioral neurogenetics (Hall, 1994; Kyriacou, 2007).

To test whether males fine‐tune their song production depending on social experience, we reared *D. melanogaster* and *Drosophila simulans* focal males either with conspecifics, with heterospecifics or in isolation, and analyzed their subsequent IPI, SSF and song production (Figure 1). *Drosophila melanogaster* and *D. simulans* are closely related, but males produce species‐specific IPIs and SSF when raised under ordinary conditions with conspecifics (Cowling & Burnet, 1981). The species identity of social partners has recently been shown to affect other male sexual behaviors in these species (Bretman, Rouse, Westmancoat, & Chapman, 2017). However, whether courtship song can be modified through social interactions with other males is at present not known. When in close proximity, male flies court and sing to other males, enage in aggressive physical contact, and can transmit visual and olfactory cues (Bailey, Hoskins, Green, & Ritchie, 2013; Griffith, 2014), so our experimental manipulation is likely to involve a full multimodal social experience (Krupp et al., 2008). In a first experiment, we tested whether male singing behavior responds to con‐ and heterospecific social environments. In a second experiment, we investigated the underlying mechanisms of song plasticity to the social environment, using neurological mutant strains that show learning and memory defects.

## 2 MATERIAL AND METHODS

Flies were reared on a 12:12 light:dark cycle at 23°C. Fly density was standardized to 10 males and 10 females per vial for at least two generations prior to sampling experimental individuals. In experiment 1, we used *D. melanogaster* Canton‐S, and *D. simulans *14021–0251.199 (San Diego stock centre). In experiment 2, we used *D. melanogaster dunce* and *amnesiac* mutants (on a Canton‐S background) that, respectively, show short‐ and middle‐term memory deficiencies (including courtship behavior) (Dubnau & Tully, 1998; Emmons & Lipton, 2003; Quinn & Dudai, 1976; Quinn, Sziber, & Booker, 1979; Rouse, Watkinson, & Bretman, 2018).

Focal males were reared either in isolation, with five conspecific males, or with five heterospecific males for 6 days (Figure 1). To visually distinguish focal males, we removed the last two or three tarsi of their left mesothoracic leg (including focals raised in isolation), from which flies rapidly recover and seem to show no significantly impaired movement. We removed the focal male without anesthetic and immediately placed it in a recording chamber for 10 min with a conspecific 1‐day‐old female. Copulation did not occur in our trials (1‐day‐old females usually do not copulate), which allowed us to record all trials for the same duration but prevented us from gathering data on mating. Recordings used an INSECTAVOX (Gorzyca & Hall, 1987) with internal temperature recording, band‐pass filtered (Fern Developments EF5‐04) at 100–700 Hz, and digitized with the software Audacity (www.audacityteam.org).

We measured the interpulse interval (IPI), the sinesong frequency, and the pulse song burst duration using DataView v.10.6.0 (Heitler, 2007). Pulse song was analyzed after filtering all song files (100–500 Hz frequency range). We isolated pulses by applying a Teager Energy Operator (time = 3, iteration = 4) and a Hill‐Valley analysis (uphill and downhill thresholds = 50%, absolute peak height filter = 15 robust *SD*, max duration = 15 ms). Then we detected pulse song bursts using specific parameters for *D. melanogaster* and *D. simulans* strains. For *D. melanogaster* strains, bursts were detected when they contained five pulse intervals of <55 ms, with an average duration of 25–50 ms and a coefficient of variation of 50%. For the *D. simulans* strain, bursts were detected when they contained five pulse intervals of <75 ms, with an average duration of 30–70 ms and a coefficient of variation of 50%. We discarded any pulses that occurred outside a burst. Finally, we averaged the interpulse intervals (IPIs), and the pulse song burst durations over the 10‐min recordings. Sinesong frequency was computed using a fast Fourier transform algorithm on manually isolated sinesong bursts (resolution <1 Hz, FFT window = Hamming, and 50% overlap).

For IPI, SSF, and pulse song burst duration, we used ANCOVAs to test the effects of focal strain, social environment, the focal strain × social environment interaction, with recording temperature as a covariate (23.7°C [19.5–26.2] and 24.3°C [21.8–26.5] in experiments 1 and 2, respectively; mean [min–max]). We conducted post hoc Tukey’s HSD tests for pairwise comparisons. Burst duration was ln transformed prior to analysis to account for skewed data distributions. Heteroscedasticity and residual distributions were checked, and Welch ANOVAs were performed when the data showed unequal variances among groups. All statistical analyses were carried out in JMP 7.0 (SAS Institute Inc., Cary NC, USA).

## 3 RESULTS

### 3.1 Experiment 1

**Figure 2:**
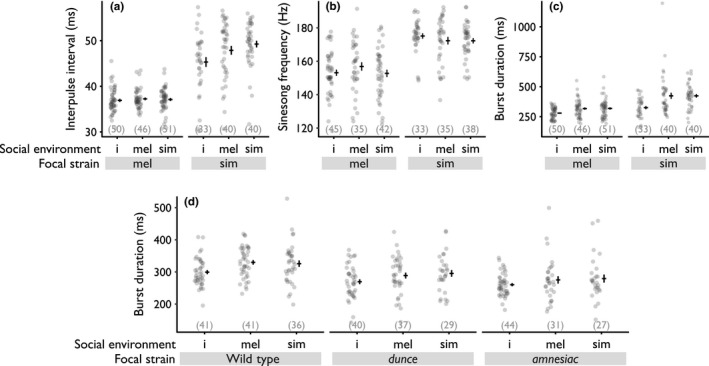
Effects of focal strains and social environment on courtship song of *Drosophila melanogaster* and *Drosophila simulans* wild‐type strains (a–c), and on *D. melanogaster* wild‐type and memory mutant strains (d). Means and 95% confidence intervals are indicated by the horizontal and vertical black bars, respectively. i: isolated, mel: D*. melanogaster*; sim: D*. simulans*. Sample sizes are indicated in brackets, and statistics are in Table 1

Isolated *D. simulans* males produced shorter IPIs than those with social experience, but in contrast, *D. melanogaster* IPI was not significantly influenced by the social environment (Figure 2a, Table 1). *Drosophila simulans* males produced longer IPIs than *D. melanogaster* males (Figure 2a, Table 1) but, because their variances differed (Figure 2a), we also used a Welch ANOVA to confirm this effect (*F* = 299.4, *df*_num_ = 1, *df*_den_ = 140.0, *p* < 0.001). SSF was not affected by the social environment in either species (Figure 2b, Table 1). Pulse song burst duration was affected by both focal strain and the social environment: Isolated males of both species produced shorter pulse song bursts compared to both the conspecific and heterospecific treatments (Figure 2c, Table 1). Note that longer pulse song duration produced by males previously reared with a social partner is not due to longer IPI, but reflects a greater number of pulses per burst (males raised in isolation produced 8.2 ± 1.3 pulses/burst, while males raised with con‐ or heterospecific males, respectively, produced 9.3 ± 1.9 and 9.5 ± 2.4 pulses/burst [mean ± *SD*]; ANCOVA: focal strain: *F*_1,253_ = 0.1, *p* = 0.74, social environment: *F*_2,253_ = 12.7, *p* < 0.001, interaction: *F*_2,253_ = 0.8, *p* = 0.43, temperature: *F*_1,253_ = 0.1, *p* = 0.75). See Supporting Information Appendix S1 for additional statistics.

**Table 1:** ANCOVAs summary statistics

	*df*_num_	*df*_den_	*F* ratio	*p* value
Experiment 1: *Drosophila melanogaster* and *Drosophila simulans*				
Interpulse interval (IPI)				
Focal strain	1	253	408.7	**<0.001**
Social environment	2	253	5.4	**0.005**
Interaction	2	253	4.7	**0.010**
Temperature	1	253	31.1	**<0.001**
Sinesong frequency				
Focal strain	1	221	125.8	**<0.001**
Social environment	2	221	0.5	0.591
Interaction	2	221	1.3	0.265
Temperature	1	221	16.8	**<0.001**
Pulse song burst duration				
Focal strain	1	253	52.9	**<0.001**
Social environment	2	253	16.3	**<0.001**
Interaction	2	253	2.5	0.088
Temperature	1	253	4.0	**0.047**
Experiment 2: *D. melanogaster* memory mutants				
Pulse song burst duration				
Focal strain	2	316	22.6	**<0.001**
Social environment	2	316	6.9	**0.001**
Interaction	4	316	0.3	0.894
Temperature	1	316	15.7	**<0.001**

Significant *p* values are indicated in bold.

### 3.2 Experiment 2

Experiment 2 confirmed that *D. melanogaster* produced longer pulse song bursts after exposure to other males (Figure 2d, Table 1). However, memory mutant strains responded to the social environment in a similar way as the wt strain, because focal strain and social experience affected pulse song burst duration but there was no significant interaction between these effects. Moreover, dunce and amnesiac males produced significantly shorter pulse song bursts than the *wt* *D. melanogaster* strain, presumably due to pleiotropic effects (Table 1). See Supporting Information Appendix S1 for additional statistics.

## 4 DISCUSSION

Social plasticity in sexual signals may have important consequences for the evolution of sexual isolation (Irwin & Price, 1999; Verzijden et al., 2012). We found that male fruit flies exposed to other males during development alter their song production. When reared in a social environment with other individuals of the same sex, males of both species produce longer song bursts (and, in *D. simulans, *slightly longer IPIs). Unexpectedly, this social effect was consistent regardless of which species the focal males experienced in previous social encounters.

Our finding of elevated courtship intensity or effort is similar to effects of social experience on mate choice described in fruit flies (Bretman, Fricke, & Chapman, 2009) and other insects (Bailey & Macleod, 2014). Males reared in social groups may expect greater risk and intensity of intrasexual competition over mating partners and therefore raise their courtship efforts upon contacting females. We are unaware of any evidence suggesting that females express preferences for burst length. However, increased burst length during courtship will result in more song per unit time, which seems likely to increase the stimulatory effect of song on females and thus the copulation success of males that produce longer bursts. Consistent with this, male *D. pseudoobscura* experimentally evolved under heightened sexual competition increase the amount of song they can sustain during active courtship (Debelle, Courtiol, Ritchie, & Snook, 2017). Also, *D. melanogaster* males produce shorter burst lengths of songs when courting females in the presence of other males (Tauber & Eberl, 2002), suggesting that males may modulate their singing efforts in opposing ways depending on whether the competition with other males is direct or not.

*Drosophila* males are known to be highly sensitive to variation in their social environment, to which they can respond by strategically allocating resources such as sperm and accessory gland secretions (Garbaczewska et al., 2013; Sirot et al., 2011; Wigby et al., 2009). In our study, males did not seem to distinguish between con‐ and heterospecific social partners as they respond to both similarly by increasing their song bout duration. This contrasts with recent findings showing that *D. melanogaster* males increase their mating duration to a lesser extent after being raised with *D. simulans* males than with *D. melanogaster* males (Bretman et al., 2017), suggesting that the response may be finely tuned to species identity for some but not all traits. Both species are commonly co‐collected with standard *Drosophila* trapping techniques and mixed species mating encounters have been described, but the likelihood and intensity of these mixed species interactions during development or courtship behavior in the field is not well understood (Gromko & Markow, 1993).

The memory mutant strains we used to investigate the role of learning in plastic song responses have been shown to display dysfunctional social responses and courtship learning (Griffith, 2014). For instance, amnesiac, but not dunce, do not increase mating duration in response to the presence of conspecific rivals (Rouse et al., 2018). Similarly, both amnesiac and dunce fail to suppress courtship efforts after unsuccessful mating attempts (Emmons & Lipton, 2003). We found that amnesiac and dunce strains adjusted their singing effort in response to the social environment in a similar manner to wild‐type *D. melanogaster*. We cannot reject the hypothesis that such social responses involve learning and memory, but our data suggest that the learning pathways disrupted by amnesiac and dunce mutations are not involved. We consider it likely that during early adult development, males are sensitive to the presence of other males in the environment and adjust allocation to courtship effort, as well as the strategic allocation they make to postmating investment. Allocation to singing effort could therefore represent more of an investment trade‐off modulated by social exposure, rather than an actively learned response. Further studies would be required to assess costs of such a trade‐off and the contribution of different sensory modalities or signals as cues in this plasticity.

Males raised with con‐ or heterospecific social partners produced courtship song with unchanged parameters, that is, similar IPI and SSF. Recent findings suggest that that *D. melanogaster* males can distinguish conspecific from *D. simulans* males (Bretman et al., 2017). This finely tuned response to species identity contrasts with the inflexibility of IPI and SSF we found. These courtship traits are of particular interest because they influence species isolation (Cowling & Burnet, 1981; Ritchie & Gleason, 1995; Ritchie et al., 1999). Theoretical models predict that, because phenotypic plasticity decouples a phenotype from its underlying genetics, socially inherited traits may undergo slower genetic evolution and therefore inhibit speciation (Price et al., 2003; Verzijden et al., 2012) or, alternatively, lead to faster speciation by facilitating more genetic drift (Lachlan & Servedio, 2004). Relatively inflexible or innate sexual traits may constitute more reliable cues for species discrimination than socially inherited traits. It remains possible that fruit fly courtship song is influenced by other components of the social environment such as the social environment experienced during the larval stages (Kim, Ehrman, & Koepfer, 1992, 1996), or male–female interactions during adulthood, but our results do not support the idea that intrasexual social experience influences song structure even though it can influence song preferences and responses (Li et al., 2018). Given the increasingly widespread evidence for plasticity of courtship and reproductive behaviors, it may be important in future studies to consider and test the conditions under which strong sexual trait canalization, rather than plasticity, is predicted and observed.

## CONFLICT OF INTEREST

None declared.

## AUTHOR CONTRIBUTIONS

LMO conceived the study, collected data, and analyzed data. LMO, NWB, MGR designed experiments. LMO (original draft), NWB, and MGR wrote the manuscript.

## DATA ACCESSIBILITY

Data available from the Dryad Digital Repository: https://doi.org/10.5061/dryad.c48sg35
