# Supplementary material for: Social effects on fruit fly courtship song
Source: Ecol Evol. 2018 Dec 10;9(1):410–6. doi: 10.1002/ece3.4759 (PMC6342107; doi:10.1002/ece3.4759)
Supplement: Supplementary file 1 [file ECE3-9-410-s001.docx]

APPENDIX 1.

#### Additional statistical analyses testing the effects of focal strains, social environment, the interaction focal strain × social environment, and the temperature affect the likelihood that courtship occurs (Nominal logistic regressions), the interpulse interval and the sine song frequency (ANCOVAs).

| **Experiment 1: *D. melanogaster* and *D. simulans*** | | | | | | |
| --- | --- | --- | --- | --- | --- | --- |
|  | **Courtship** | | ***df*** | ***N*** | ***χ^2^*** | ***P* value** |
|  |  | focal strain | 1 | 361 | 28.0 | **<0.001** |
|  |  | social environment | 2 | 361 | 1.6 | 0.459 |
|  |  | interaction | 2 | 361 | 2.0 | 0.365 |
|  |  | temperature | 1 | 361 | 0.6 | 0.439 |
| **Experiment 2: *D. melanogaster* memory mutants** | | | | | | |
|  | **Courtship** | | | | | |
|  |  | focal strain | 2 | 430 | 8.9 | **<0.001** |
|  |  | social environment | 2 | 430 | 45.8 | **<0.001** |
|  |  | interaction | 4 | 430 | 11.1 | **0.025** |
|  |  | temperature | 1 | 430 | 1.5 | 0.214 |
|  | **Interpulse interval (IPI)** | | ***df*_num_** | ***df*_den_** | ***F* ratio** | ***P* value** |
|  |  | focal strain | 2 | 316 | 432.5 | **<0.001** |
|  |  | social environment | 2 | 316 | 8.7 | 0.332 |
|  |  | interaction | 4 | 316 | 9.5 | 0.662 |
|  |  | temperature | 1 | 316 | 650.0 | **<0.001** |
|  | **Sine song frequency** | | | | | |
|  |  | focal strain | 2 | 280 | 14.3 | **<0.001** |
|  |  | social environment | 2 | 280 | 12.8 | **<0.001** |
|  |  | interaction | 4 | 280 | 1.4 | 0.235 |
|  |  | temperature | 1 | 280 | 9.8 | **0.002** |
